# Ketogenic diet in pyruvate dehydrogenase complex deficiency: short- and long-term outcomes

**DOI:** 10.1007/s10545-016-0011-5

**Published:** 2017-01-18

**Authors:** Kalliopi Sofou, Maria Dahlin, Tove Hallböök, Marie Lindefeldt, Gerd Viggedal, Niklas Darin

**Affiliations:** 1 Department of Pediatrics, The Queen Silvia Children’s Hospital, University of Gothenburg, Smörslottsgatan 1, 41685 Gothenburg, Sweden; 2 Department of Pediatrics, Astrid Lindgren Children’s Hospital, Karolinska Institute, Stockholm, Sweden

## Abstract

**Objectives:**

Our aime was to study the short- and long-term effects of ketogenic diet on the disease course and disease-related outcomes in patients with pyruvate dehydrogenase complex deficiency, the metabolic factors implicated in treatment outcomes, and potential safety and compliance issues.

**Methods:**

Pediatric patients diagnosed with pyruvate dehydrogenase complex deficiency in Sweden and treated with ketogenic diet were evaluated. Study assessments at specific time points included developmental and neurocognitive testing, patient log books, and investigator and parental questionnaires. A systematic literature review was also performed.

**Results:**

Nineteen patients were assessed, the majority having prenatal disease onset. Patients were treated with ketogenic diet for a median of 2.9 years. All patients alive at the time of data registration at a median age of 6 years. The treatment had a positive effect mainly in the areas of epilepsy, ataxia, sleep disturbance, speech/language development, social functioning, and frequency of hospitalizations. It was also safe—except in one patient who discontinued because of acute pancreatitis. The median plasma concentration of ketone bodies (3-hydroxybutyric acid) was 3.3 mmol/l. Poor dietary compliance was associated with relapsing ataxia and stagnation of motor and neurocognitive development. Results of neurocognitive testing are reported for 12 of 19 patients.

**Conclusion:**

Ketogenic diet was an effective and safe treatment for the majority of patients. Treatment effect was mainly determined by disease phenotype and attainment and maintenance of ketosis.

**Electronic supplementary material:**

The online version of this article (doi:10.1007/s10545-016-0011-5) contains supplementary material, which is available to authorized users.

## Introduction

The ketogenic diet (KD) was first introduced as an antiepileptic treatment, in 1921 (Wilder 1921), but it was not until 1976 that KD was shown to be beneficial in pyruvate dehydrogenase complex (PDC) deficiency (Falk et al. 1976). The biochemical rationale behind the metabolic effect of KD lies in the fact that this is a high-fat, low-carbohydrate diet that mimics the metabolic state of long-term fasting. In PDC deficiency, the glycolytic end product, pyruvate, is not optimally metabolized through the tricarboxylic acid cycle, leading to increased production of lactate and impaired production of adenosine triphosphatase (ATP) via the mitochondrial respiratory chain (Reed 1974; Robinson et al. 1987). During carbohydrate deprivation, cellular energy is no longer derived from glycolysis but from degradation of fatty acids. The ketone bodies (3-beta-hydroxybutyrate, acetoacetate, and acetone) generated by fatty acid oxidation serve as an alternative energy substrate to glucose for the brain (Pierre and Pellerin 2005; Scholl-Bürgi et al. 2015).

In this study, we present the results of a Swedish cohort of 19 pediatric patients with PDC deficiency treated with KD. The aim was to depict the short- and long-term effects of KD on disease course and disease-related outcomes and elucidate metabolic factors associated with these outcomes. Potential safety and compliance issues related to this dietary treatment were also evaluated.

## Methods

### Setting

This was a longitudinal cohort study of pediatric patients diagnosed with PDC deficiency in Sweden and treated with KD between January 2009 and March 2016. All patients diagnosed and followed according to a specific protocol (supplementary Table e1) at the two referral centers for inherited metabolic diseases in Sweden—the Sahlgrenska University Hospital in Gothenburg and the Karolinska University Hospital in Stockholm—were included.

### Inclusion criteria

Inclusion criteria were:

(1) Disease onset before 18 years of age

(2) Clinical and laboratory phenotype compatible with PDC deficiency

(3) Presence of pathogenic mutation(s) associated with PDC deficiency

(4) Treatment with KD for a minimum of 3 months

(5) Data availability on medical records to achieve >90% of data point coverage on the Case Report Form (CRF).

### Data analysis

Patients were prospectively evaluated at baseline then 3, 6, and 12 months, and then every sixth month according to the ketogenic diet protocol (Table e1). Each patient was clinically assessed by the same physician across evaluations. Neurocognitive development was assessed at baseline, 6 months, 12 months, and every second year with the help of the Griffiths Mental Development Scales and the Wechsler Scales for Children (WPPSI-III and WISC-IV), where appropriate, depending on patient’s chronological age and cognitive development at baseline. The main neurocognitive outcomes were speech and language development, performance, social functioning, and composite score of the neurocognitive subtests, indicating patient intelligence. The latter is presented as a full-scale intelligence quotient (IQ) or general developmental quotient (GQ).

Gross- and fine-motor development was assessed by the pediatric neurologist and physiotherapist who followed the patient. Dietary compliance was assessed from: (a) ketosis level based on the levels of 3-hydroxybutyric acid (ketone bodies) measured at home with a hand-held meter and at the laboratory at follow-up visits; (b) nutrient intake, estimated with the help of 4-day food records collected at each follow-up visit. Safety outcomes were assessed with the help of regular monitoring of side effects and biochemical investigations performed in fasting state (Table e1).

The overall treatment outcome was assessed at the last follow-up visit and compared with baseline using the Clinical Global Impression of Improvement (CGI-I) scale, ranging from 1 (very much improved) to 7 (very much worse). At the last follow-up visit, parents were asked to complete a questionnaire sharing their perspectives of global improvement from baseline to last follow-up visit. Parents were also asked to evaluate their child’s improvement during treatment from 1 (very much improved) to 7 (very much worse) in the following areas: motor skills, cognitive skills, social/behavioral skills, and epileptic seizures. Neuroradiologic examination before and after KD initiation was not part of the protocol.

All data were retrospectively collected from patient records with the help of a CRF. Since PDC deficiency is a rare neurometabolic disorder, the statistical methods applied were mainly descriptive. The two-tailed Wilcoxon signed-rank test was used to compare lactate values before versus after KD start. The two-tailed paired *t* test was used to compare morning versus evening lactate levels. A *p* value <0.05 was considered significant.

### Systematic literature review

Study methodology is presented in Table e2.

### Ethical considerations

The study was approved by the Regional Research Ethics Committee at the University of Gothenburg, Sweden.

## Results

### Perinatal history

A total of 19 patients (3 boys, 16 girls) were assessed, the majority (15/19) having prenatal disease onset based on clinical and/or neuroimaging grounds. Complications of pregnancy occurred in four cases: pre-eclampsia (2), oligohydramnios (1), and gestational diabetes mellitus (1). All patients were born at term; intrauterine growth restriction was present in two. Disease manifestations were present at birth or occurred soon afterward in ten patients. These diseases were mainly microcephaly (7), congenital lactic acidosis (7), muscular hypotonia and feeding difficulties (4), hypothermia (2), and hypoglycemia (1).

### Ketogenic diet

Median age at KD initiation was 2.5 years (week 1 of life to15 years, 3 months). Seven patients received classic KD and 12 modified KD (MKD), i.e. KD, with lower ketogenic ratio; the vast majority received ketogenic booster (Table 1). Follow-up was a median of 2.9 years (6 months to 6 years,11 months); all were alive at the time of data registration, at a median age of 6 years (2 years to 16 years 5 months). All but two patients were started on lower ratios up to 2.5:1 (fat:carbohydrate plus protein) and were changed to higher ratios ≥6 months.

**Table 1:** Genotype, phenotype, dietary composition, lactate, current status, compliance, and CGI-I (*n* = 19)

ID no.	Gender	Gene	Onset	Predominant clinical phenotype	Predominant MRI findings	Age at diagnosis	Age at KD start	Type of KD	KD duration	KD ratio start vs last	Carbohydrates (g/d) start vs last	Daily calories per kg	B-lactate prior / last follow-up	Current status	Poor compliance	CGI-I
1	F	*PDHA1*	Prenatal	IUGR, CLA, psychomotor delay, epilepsy (after KD start)	CC agenesis	0 months	0 months	Classic	2 years 7 months	1,5:1 3.0:1	10.9 6.3	51	11/ 1	Ongoing	No	Much worse
2	M	*PDHA1*	Prenatal	CLA, psychomotor delay, metabolic stroke	CC dysgenesis, white matter atrophy, metabolic stroke lesions	3 months^a^/7 months	7 months	Classic	5 years 9 months	1.5:1 3.0:1	19 12	81	12/ 2.1	Ongoing	No	Much improved
3	F	*PDHA1*	Prenatal	Psychomotor delay	White matter atrophy	1 year 6 months	1 year 9 months	Classic	6 months	2.0:1 2.0:1	20 20	77	2.7/ 1.9	Ongoing	No	Much improved
4	F	*PDHA1*	Prenatal	Psychomotor delay, spasticity, epilepsy	CC dysgenesis	14 years 10 months	15 years 3 months	Classic	1 years 2 months	2.5:1 3.0:1	23 10	40	2.1/ 1.7	Ongoing	No	Very much improved
5	F	*PDHA1*	Prenatal	Psychomotor delay, spasticity, epilepsy	CC dysgenesis, white matter atrophy	8 months	9 months	Classic	3 years 6 months	2,0:1 2.5:1	15 5	70	4/ 1.9	Ongoing	No	Much improved
6	F	*PDHA1*	Prenatal	CLA, psychomotor delay, spasticity, ataxia	White matter atrophy	14 years 1 month	14 years 5 months	Classic	10 months	3,0:1 3.0:1	12 8	36	4.7/ 3.5	Ongoing	No	Much improved
7	F	*PDHA1*	Prenatal	CLA, psychomotor delay, epilepsy	CC dysgenesis, white matter atrophy	1 month	1 month	Classic	2 years 6 months	1.5:1 3.0:1	10.9 7.5	65	2.3/ 1.5	Ongoing	No	Minimally improved
8	F	*PDHX*	Prenatal	IUGR, psychomotor delay, epilepsy	CC dysgenesis	9 years 1month	9 years 6months	MKD	2 years 10 months	1.5:1 4.0:1	40 7.5	36	1/ 2	Ongoing	Yes	Minimally improved
9	F	*PDHA1*	Childhood	Psychomotor delay, episodic ataxia	Leigh syndrome	5 years	8 years	MKD	6 years 2 months	2.2:1 2.5:1	26 15	48	1.3/ 0.6	Ongoing	Yes	Minimally improved
10	F	*PDHA1*	Prenatal	CLA, psychomotor delay, spasticity, epilepsy	CC agenesis, white matter atrophy	3 years 8 months	4 years 2 months	MKD	1 year 3 months	2.2:1 2.1:1	40 20	55	3/ 2.2	discontinued	No	Much improved
11	M	*PDHA1*	Childhood	Episodic ataxia	Normal	2 years 5 months	3 years 5 months	MKD	6 years 11 months	1.0:1 NA	45 NA	77	1.4/ 1.5	Ongoing	No	Much improved
12	F	*PDHA1*	Prenatal	Psychomotor delay, hearing impairment, epilepsy	White matter atrophy	1 year 5 months	1 year 7 months	MKD	5 years 5 months	3.0:1 4.0:1	14 6	60	2.9/ 3	Ongoing	No	Much improved
13	F	*PDHA1*	Prenatal	Psychomotor delay, ataxia	White matter atrophy	2 years 6 months	2 years 8 months	MKD	4 years	1.3:1 3.0:1	30 9	40	1.5/ 2	Ongoing	No	Very much improved
14	F	*PDHA1*	Prenatal	Psychomotor delay, ataxia	White matter atrophy	1 year 7 months	2 years	MKD	3 years 7 months	1.8:1 3.0:1	40 20	117	1.3/ 1.4	Ongoing	No	Very much improved
15	F	*PDHA1*	Prenatal	CLA, psychomotor delay, spasticity, epilepsy	CC agenesis	1 year 2 months	1 year 4 months	MKD	3 years	1.5:1 4.0:1	20 3	49	3.4/ 1	Ongoing	No	Minimally improved
16	F	*PDHA1*	Prenatal	Psychomotor delay, spasticity, dystonia, epilepsy	CC dysgenesis, white matter atrophy	8 years 2 months	3 years 9 months	MKD	5 years 9 months	2.0:1 4.0:1	13 5	65	NA/ 1.6	Ongoing	No	Minimally improved
17	M	*PDHX*	Prenatal	CLA, psychomotor delay, epilepsy	White matter atrophy	1 month^a^/ 6 years 2 months	5 years 6 months	MKD	2 years 10 months	1.5:1 3.0:1	40 8.5	71	2.1/ 1.7	Ongoing	No	Much improved
18	F	*PDHA1*	Infantile	Psychomotor delay, episodic ataxia	Normal	5 years 9 months	6 years	MKD	2 years 4 months	1.5:1 2.5:1	30 15	57	2.7/ 1.8	Ongoing	No	Much improved
19	F	*PDHA1*	Infantile	Psychomotor delay, epilepsy, poststroke hemiparesis	Unilateral stroke lesions	9 months	1 year	MKD	2 years 4 months	1.5:1 2.5:1	30 15	58	2/ 1.6	Ongoing	No	Minimally improved

*M* male, *F* female, *IUGR* intrauterine growth restriction, *CLA* congenital lactic acidosis, *CC* corpus callosum, *KD* ketogenic diet, *MKD* modified ketogenic diet, *CGI-I* Clinical Global Impression of Improvement, *PDC* pyruvate dehydrogenase complex

^*^Age at diagnosis in patients 2 and 17: first age refers to diagnosis based on enzyme activity and second to genetic confirmation of PDC deficiency

### Clinical efficacy

Ten patients developed epilepsy before beginning the diet; seven had generalized epilepsy (2 with West syndrome), and the remaining three had focal epilepsy with seizures occurring on a daily basis. Nine patients had abnormal electroencephalogram (EEG) prior to starting the KD, as follows: abnormal background (8), epileptiform activity (5), and hypsarrhythmia (1); two patients, one with poststroke seizures and another with focal epilepsy, became seizure free on single antiepileptic medication before beginning the diet. KD had a positive effect on epilepsy in all patients; in half, seizures disappeared within 1 year. Follow-up EEG was available in five patients and showed improvement in four (completely normal in 1) and worsening in one. One patient with focal epilepsy before KD became seizure free but had to discontinue the diet due to side effects. After KD discontinuation, the epilepsy relapsed, and follow-up EEG showed increased epileptiform activity.

Six patients had ataxia initially, characterized by worsening symptom severity with infections (6/6) and carbohydrate deficiency (1/6); two (patients 9 and 11) had childhood-onset ataxia with Leigh syndrome phenotype (patient 9) and episodic ataxia (patient 11). The diet positively affected ataxia in all patients by increasing remission periods and decreasing severity and duration. The effect was sustained during infections.

Eight of 19 patients suffered from sleep disturbance prior to KD characterized by at least one of the following: difficulty falling asleep, fragmented nocturnal sleep, early-morning awakening, and excessive daytime somnolence. The diet was effective on nocturnal awakenings and excessive daytime somnolence, but the majority of patients (6/8) still required medication to help them fall asleep.

Brain magnetic resonance imaging (MRI) was performed in five patients at various time points before and after initiation of KD. No correlation between MRI findings and treatment outcomes could be established.

### Neurodevelopmental outcomes

**Fig. 1:**
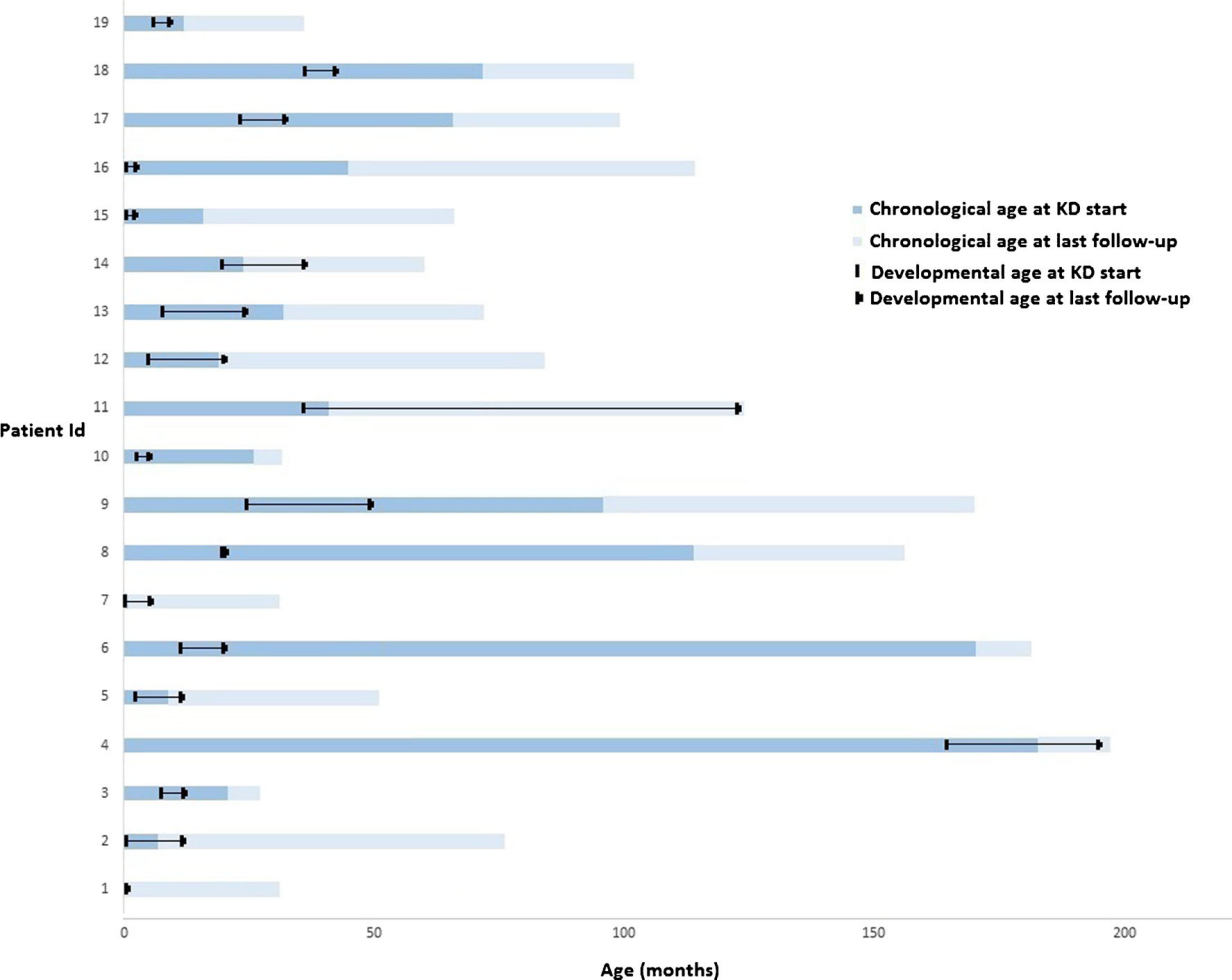
Gross motor development at baseline and during treatment (*n* = 19)

A delay in early motor milestones was observed in most patients. At baseline, the median developmental age of gross and fine motor skills were 8 (Q1: 0 months; Q4: 2 years) and 7.5 (Q1: 0 months; Q4: 2 years) months, respectively. The best motor development during treatment was seen in two patients (9 and 11) with childhood-onset ataxia. Six patients had spasticity at baseline, with no change during treatment. Motor development from baseline to last follow-up for each patient separately is shown as developmental versus chronological age (Figs. 1 and e2).

**Fig. 2:**
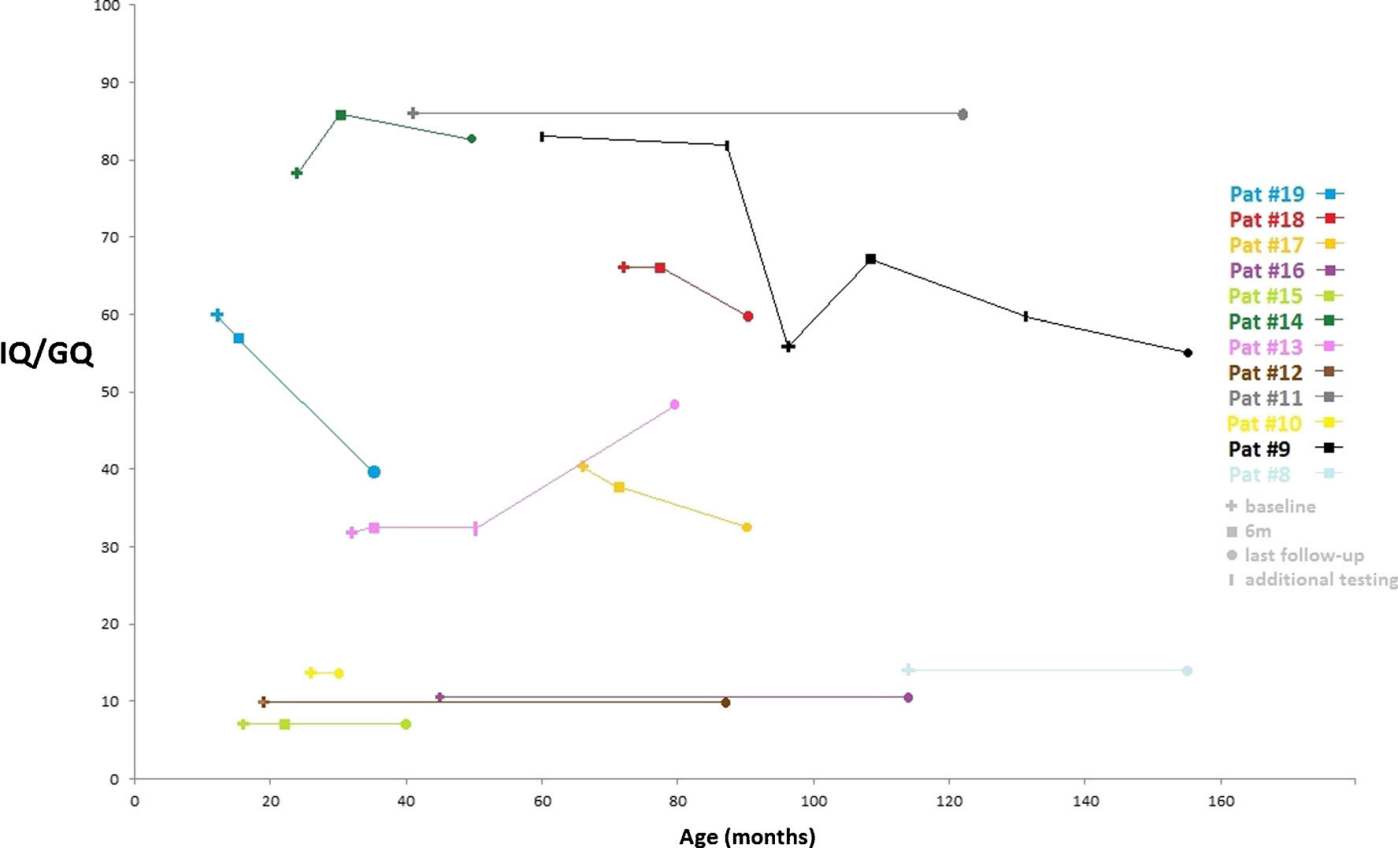
Full-scale intelligence quotient (IQ) or general developmental quotient (GQ) at baseline and during treatment (*n* = 12).

**Fig. 3:**
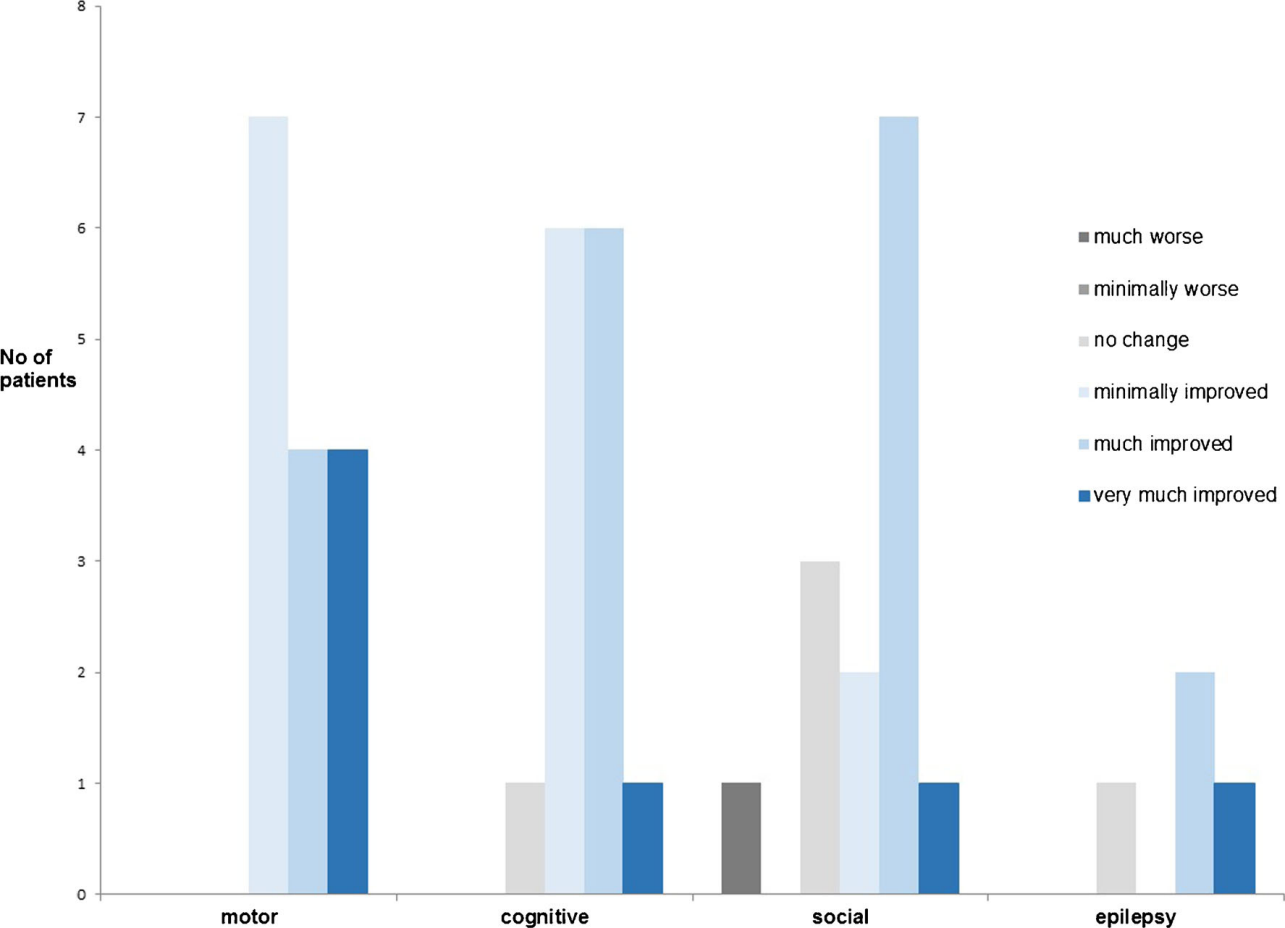
Parental impression of patient’s global improvement from baseline to last follow-up (*n* = 15)

Minimal or no acquisition of gross motor skills was seen in three patients (1, 15, 16) who had no motor skills at baseline and reached a maximum developmental age of 3 months at last follow-up; one patient (8) with gross motor development of 20 months did not acquire new gross motor skills. All remaining patients presented slow but positive development of both gross and fine motor skills during dietary treatment. Improvement in speech and language was seen in 15 of 19 patients during KD treatment. Patients who developed mostly in speech and language were the two with childhood-onset ataxia and dysarthria (9, 11), and one patient with infantile-onset disease with dysarthria (18). At last follow-up, patients 11 and 18 had normal speech and language development with very mild dysarthria. Patient 9 had slightly delayed speech with mild dysarthria. This patient’s verbal IQ developed from 69 at baseline to 77 1 year after KD, to 73 after 2 years, and has been stable at 65 the last 4 years. This patient had periods with poor dietary compliance accompanied by worsening dysarthria and stagnation of speech development. Three patients (13, 14, 17) developed language skills to a minimum language developmental age of 2.5 years at last follow-up. Slow but positive development during treatment was seen in nine patients, from no speech at baseline to producing sounds, letters, or a couple of words up to a developmental age of 15 months. Two patients developed no speech (1, 16); there was no change in speech or language skills during treatment in patients 8 and 12, the latter of whom also had severe hearing impairment.

Patients’ behavioral and social functioning was evaluated clinically by the neurologist—and a neuropsychologist and/or neuropsychiatrist if required—along with parental input. All but one patient with minimal or no social skills at baseline became more active during treatment, started making eye contact and seeking attention, and were considered by their parents as more alert and less somnolent. The remaining patients who had developed social skills at baseline maintained or improved those skills over time. Three patients exhibited hyperactivity–impulsivity with or without aggression at baseline (4, 8, 17), which improved during treatment in patients 4 and17 only.

Twelve of 19 patients underwent neurocognitive testing at baseline followed by regular neurocognitive monitoring during treatment. Composite scores derived from neurocognitive testing at baseline and during treatment are presented as full-scale IQ or GQ in Fig. 2. Five of these patients had profound mental retardation at baseline with unchanged GQ during treatment. Four patients (9, 11, 13, 14) showed improved or unchanged cognitive skills over time. In patient 9, cognitive development was followed from 3 years prior to baseline to 5 years after KD initiation. This patient had a dramatic decline in full-scale IQ from 83 at 5 years to 56 at 8 years, which—in addition to worsening ataxic symptoms—motivated the family to initiate treatment with KD. One year after KD start, the patient’s full-scale IQ had increased from 56 to 67 and remained stable throughout the second year of treatment, followed by a slow decline during the next 4 years of treatment to a full-scale IQ of 55, which was the same as baseline IQ. This neurocognitive decline after 2 years of treatment was associated with poor dietary compliance. Three patients (17, 18, 19) showed decline in full-scale IQ/GQ from baseline to last follow-up (Fig. 2).

### Lactate levels, safety, and compliance

Increased blood lactate >2 mmol/l was found in 12 of 18 patients (67%) at baseline as opposed to four of 19 at last follow-up (21%) (*p* = 0.01). Side effects were observed in 13 of 19 patients: obstipation (10/13; 2/13 showed no change prior versus after beginning KD), pancreatitis (1/13), sialorrhea (1/13), and vomiting (1/13). Biochemical investigations were normal in all but one patient (Fig. e4). Patient 10 had mildly elevated transaminases at 6 months after diet initiation of no apparent cause; 15 months, the patient was admitted to the hospital with acute pancreatitis, with subsequent KD discontinuation.

According to parental log books, the majority of patients had plasma ketone levels (3-hydroxybutyric acid) >2 mmol/l; morning levels were significantly lower than evening, with a median difference of 1.4 mmol/l (0.4–2.6 mmol/l; *p* < 0.001). Two patients had compliance problems (8, 9); both had periods of inadequate ketosis despite dietary adjustments, resulting in plasma ketone levels <2 mmol/l in consecutive measurements. Patient 9 achieved good ketosis during the first 2 years but later had intermittently poor compliance, with ketone levels <2 mmol/l and parallel worsening in clinical and neurocognitive outcomes.

### Concomitant medications

Patients received the following cofactors and electrolyte substitutions: thiamine (18/19), carnitine (17/19), potassium citrate (13/19), calcium (8/19), vitamin D_3_ (7/19), magnesium (3/19), phosphorus (2/19), and dichloroacetate (2/19). Patients received 8.5–30 mg/kg per day of thiamine, and no beneficial effect could be seen by the treating physician or parents in any patient. Laxatives for obstipation (10/19), antiepileptics (6/19), and melatonin to induce sleep (5/19) were also used.

### Parent and investigator impression of improvement

As shown in Table 1, 18 of 19 patients were considered by the investigators as improving at least minimally from baseline to last follow-up. Parental impression of the dietary effects on motor function, cognition, social/behavioral skills, and epilepsy is demonstrated in Fig. 3. Most parents considered their children as being much improved in cognitive and behavioral/social functioning after diet initiation. At last follow-up visit, parents were asked at what plasma ketone levels their children achieved optimal functioning. Parents of eight patients replied, considering optimal plasma ketone levels as being >2 mmol/l, with a median of 3.3 (2.1–4.5) mmol/l. Almost all patients who were considered by investigators as being at least much improved had ketone levels of ∼3–3.5 mmol/l, as opposed to the remaining patients, with ketone levels <3 or >4 mmol/l.

### Systematic literature review

Results from the systematic literature review are summarized in Table e2.

## Discussion

We evaluated the effect of KD treatment in 19 pediatric patients with PDC deficiency and found it effective and safe for the majority. Based on our results, KD efficacy is determined by two major variables: PDC deficiency phenotype, and attainment and maintenance of ketosis. Patients with the most positive treatment outcomes were those with infantile or childhood disease onset. These patients experienced positive effects both clinically, i.e., in epilepsy, ataxia, and sleep disturbance, and in the development of motor and neurocognitive functioning. KD was less beneficial in patients with prenatal onset and minimal functioning upon diet initiation. In these patients, structural brain abnormalities began in utero, with permanent brain damage leading to profound mental retardation and spasticity, which cannot be reversed. These patients experienced positive treatment effects mostly in the areas of epilepsy, sleep, and social functioning.

The effect of KD in intractable epilepsy is well established, with half of the treated patients achieving >50% reduction in seizure frequency by 6 months of treatment (Hallböök et al. 2015). In our study, all patients with epileptic seizures at baseline improved during KD treatment. In half of them, seizures disappeared within 1 year after diet initiation.

The effect of KD on neurocognitive and social functioning in PDC deficiency has not previously been assessed. In therapy-resistant epilepsy, treatment with KD has been associated with increased alertness and improved behavioral outcomes (Hallböök et al. 2007, 2012; Nordli et al. 2001; Pulsifer et al. 2001). Overall neurocognitive development was evaluated by the neuropsychologist as being at least minimally improved (11/12) in our study. The cognitive decline seen in patients 17, 18, and 19; Fig. 2) was not indicative of cognitive impairment during treatment, as these patients did not lose previously acquired skills but, on the contrary, continued to develop cognitively during treatment, albeit at a lower rate than expected for their biological age. As PDC deficiency is a progressive neurological disease, untreated patients would be expected to lose previously acquired neurocognitive skills over time. We believe that treatment with KD counteracts this progressive neurodegeneration.

Our patients were alive at data registration, at a median age of 6 years. This is opposed to the poor survival outcomes shown in previous studies (DeBrosse et al. 2012; Patel et al. 2012; Wexler et al. 1997). The effect of KD on survival was investigated by DeBrosse et al., and no significant correlation was found (DeBrosse et al. 2012). Our patients survived considerably longer than those in previous studies, which could be partly explained by the fact that they were mostly girls, as girls with early-onset disease survive longer than boys (DeBrosse et al. 2012); dietary treatment may also have contributed. However, the extent to which KD contributes longer survival is difficult to assess in our study or others due to lack of a control group.

Ketosis is considered to improve lactate levels in PDC deficiency (Falk et al. 1976). This is also shown in our study, as blood lactate significantly declined after KD initiation. In order to be effective, KD treatment must lead to adequate and sustained ketosis. Wexler et al. proposed the level of ketosis as the most important variable in determining outcome of patients with PDC deficiency (Wexler et al. 1997). Two patients in our study (8, 9) experienced periods of poor dietary compliance, which we consider to explain why patient 8 developed no new gross motor or language skills during treatment or improved in behavioral functioning. Patient 9 had periodically poor compliance, which was linked to episodic reappearance of ataxia and a slight cognitive decline.

To our knowledge, this is the first longitudinal study on long-term efficacy and safety outcomes of KD in patients with PDC deficiency. Based on our study, we propose that KD be introduced as early as possible upon diagnosing PDC deficiency, as early initiation may prevent further metabolic damage to the brain. The long-term effectiveness is highly dependent upon regular monitoring of plasma ketone levels and adjusting dietary composition to establish and sustain ketosis.

## Electronic supplementary material

Below is the link to the electronic supplementary material.

Table e1 Study Flowchart (DOCX 14 kb)

Table e2 Results from the systematic literature review of ketogenic diet in the treatment of PDC deficiency (DOCX 16 kb)

Figure e2 Fine-motor development at baseline and during treatment (*n* = 19). Patient 17: At last follow-up, the patient showed improvement in executive, social, and behavioral functioning but no change in language skills. Overall neurocognitive development from baseline to last follow-up was minimally improved. Patient 18 was tested with Wechsler Scales for Children (WPPSI-III) at baseline, 6 months, and 1.5 years after diet initiation. Full-scale and verbal intelligent quotient (IQC), respectively, were as follows: 66, 66, 60 (full-scale IQ) and 78, 78, 83 (verbal IQ), with improvement in language ability, processing speed, and attention over time. Overall neurocognitive development from baseline to last follow-up was minimally improved. Patient 19: At last follow-up, the patient had developed slightly in language and social skills, with the latter corresponding to a developmental age of 19–20 months old. Overall neurocognitive development from baseline to last follow-up was minimally worse (JPG 74 kb)

Figure e4 Mean values of 3-hydroxybutyric acid, cholesterol, triglycerides, high-density lipoprotein (HDL), and low-density lipoprotein (LDL) in plasma at the following time points: before diet initiation (baseline), at 6 months, at 1 year, and at last follow-up (*n* = 19) (JPG 63 kb)
