# Supplementary material for: Ketogenic diet in pyruvate dehydrogenase complex deficiency: short- and long-term outcomes
Source: J Inherit Metab Dis. 2017 Jan 18;40(2):237–45. doi: 10.1007/s10545-016-0011-5 (PMC5306430; doi:10.1007/s10545-016-0011-5)
Supplement: Supplementary file 1 — Study Flowchart (DOCX 14 kb) [file 10545_2016_11_MOESM1_ESM.docx]

| **Table e1: Study Flowchart** | | | | | | | | | | |
| --- | --- | --- | --- | --- | --- | --- | --- | --- | --- | --- |
| **Assessment per time-point** | **Baseline^1^** | **KD start** | **3 months** | **6 months** | **9 months** | **12 months** | **18 months** | **24 months** | **every 6th month from this point forward** | **End-of-study visit** |
| **Inclusion criteria** | **X** | **X** |  |  |  |  |  |  |  |  |
| **Informed consent** | **X** |  |  |  |  |  |  |  |  |  |
| **Neurologic examination** | **X** | **X** | **X** | **X** | **X** | **X** | **X** | **X** | **X** | **X** |
| **Assessment of motor and cognitive development by the investigator** | **X** | **X** | **X** | **X** | **X** | **X** | **X** | **X** | **X** | **X** |
| **Growth assessment** | **X** | **X** | **X** | **X** | **X** | **X** | **X** | **X** | **X** | **X** |
| **Neurocognitive test^2^** | **X** |  |  | **X** |  | **X** |  | **X** | **X^4^** |  |
| **Neuropsychiatric test^2^** | **X** |  |  |  |  |  |  | **X** |  |  |
| **Seizure log** | **X** | **X** | **X** | **X** | **X** | **X** | **X** | **X** | **X** | **X** |
| **4-day dietary log** | **X** |  | **X** | **X** | **X** | **X** | **X** | **X** | **X** | **X** |
| **Ketone log** | **X** | **X** | **X** | **X** | **X** | **X** | **X** | **X** | **X** | **X** |
| **Biochemical investigations^3^** | **X** | **X** | **X** | **X** | **X** | **X** | **X** | **X** | **X** | **X** |
| **CGI-I** |  |  | **X** | **X** | **X** | **X** | **X** | **X** | **X** | **X** |
| **Sleep-EEG** | **X** | **X** |  |  |  | **X** |  |  |  |  |
| **Parental questionnaire** |  |  |  | **X** |  | **X** |  | **X** |  | **X** |
| **Concomitant medications** |  | **X** | **X** | **X** | **X** | **X** | **X** | **X** | **X** | **X** |
| **Adverse events** |  |  | **X** | **X** | **X** | **X** | **X** | **X** | **X** | **X** |
| **KD: Ketogenic diet; CGI-I: Clinical global impression of improvement (assessed by the investigator)**  **^1^ Baseline: Up to 3 months before initiation of KD**  **^2^ Neurocognitive and neuropsychiatric testing is performed on these specific time-points in patients followed at the Sahlgrenska University Hospital**  **^3^ Biochemical investigations are performed in fasting state and include (in blood): lactate, ketone bodies, venous blood gases, complete blood count, transaminases, alkaline phosphatase, albumin, gamma-GT, bilirubin, electrolytes, creatinine, urea, uric acid, iron, TIBC, transferrin, ferritin, LD, triglycerides, HDL, LDL, cholesterol, carnitine and acylcarnitines**  **^4^ Neurocognitive test: After 24 months, the neurocognitive testing is performed every second year.** | | | | | | | | | | |
