# Supplementary material for: Ketogenic diet in pyruvate dehydrogenase complex deficiency: short- and long-term outcomes
Source: J Inherit Metab Dis. 2017 Jan 18;40(2):237–45. doi: 10.1007/s10545-016-0011-5 (PMC5306430; doi:10.1007/s10545-016-0011-5)
Supplement: Supplementary file 2 — Results from the systematic literature review of ketogenic diet in the treatment of PDC deficiency (DOCX 16 kb) [file 10545_2016_11_MOESM2_ESM.docx]

| **Table e2: Methodology and results from the systematic literature review of the ketogenic diet in the treatment of pediatric patients with pyruvate dehydrogenase complex deficiency** | | | | | | | | | | |
| --- | --- | --- | --- | --- | --- | --- | --- | --- | --- | --- |
| **Methodology:**  All published cases (including case reports, case series and clinical studies) referring to the effect of KD on pediatric patients with PDC deficiency were included. These were grouped according to the following criteria:  **Group A**: Case reports or case series of pediatric patients with PDC deficiency and available data on (i) underlying genetics, (ii) KD start and/or duration, (iii) type of KD and (iv) treatment outcomes.  **Group B**: Case reports or case series lacking at least one of the above mentioned criteria.  **Group C**: Reports on the effect of KD as part of a larger study on PDC deficiency, lacking specific dietary or outcome data for the treated patients. | | | | | | | | | | |
| **Group A** | | | | | | | | | | |
| **Publication** | **No of patients (M;F)** | **Genetics** | **Age at KD start** | **Duration on KD** | **Type of KD** | **Survival** | **Efficacy outcomes** | **Safety outcomes** | **Compliance outcomes** | **KD effective/ ineffective** |
| **Wexler et al, 1997** | 7 (7M) | *PDHA1* | 1y (range: 2w-11y) | 3y4m (range: 3.25m-14y4m) | Variable | All died at a median age of 3y5m (1y1m-16y) | Survival, max developmental age, blood lactate | Ketoacidosis | Ketone levels | Effective when administered early |
| **Prasad et al, 2011** | 2 (1M;1F) | *PDHB*(M); *PDHA1*(F) | 6m (M); 1y5m (F) | 6.5y (M); 3y7m (F) | 80%fat-10%carbo(M); 65%fat-19%carbo(F) | Both alive 7y(M);5y(F) | Survival, disease course/hospitalizations, neurodevelopmental outcomes | None reported | None reported | Effective |
| **Wada et al, 2004** | 1 (1F) | *PDHA1* | 13m | 3m | high-fat | Unknown | Disease course | None reported | Unknown | Ineffective |
| **Wijburg et al, 1992** | 1 (1M) | *PDHA1* | 1y | 2y | 58%fat-31%carbo | Alive at 3y | Survival, disease course/hospitalizations, neurodevelopmental outcomes, follow-up MRI, blood lactate | Ketoacidosis | Ketone levels | Effective |
| **El-Gharbawy et al, 2011** | 1 (1M) | *PDHA1* | 1y3m | 2y | 4:1-3:1-MDK | Aliveat 3y3m | Survival, disease course/hospitalizations, neurodevelopmental outcomes, blood lactate | None reported | Dietary non-compliance on classical KD | Effective |
| **Sharma et al, 2011** | 1 (1F) | *PDHA1* | 1y1m | 5m | 2.0:1 | Alive at 1y6m | Survival, disease course/hospitalizations, neurodevelopmental outcomes | Well tolerated | None reported | Effective |
| **Group B** | | | | | | | | | | |
| **Publication** | **No of patients (M;F)** | **Available data** | **Missing data** | **KD effective/ ineffective** |  |  |  |  |  |  |
| **Falk et al, 1976** | 2 (2M) | KD and treatment outcomes | Genetics | Effective |  |  |  |  |  |  |
| **Steller et al, 2013** | 1 (1M) | Genetics, type of KD, survival, treatment outcomes | KD start or duration | Effective |  |  |  |  |  |  |
| **Endo et al, 1989** | 1 (1M) | Genetics, disease course | KD type, start, duration | Unknown |  |  |  |  |  |  |
| **Singhi et al, 2013** | 1 (1M) | Genetics, disease course | KD type, start or duration, no treatment outcomes | Effective |  |  |  |  |  |  |
| **Di Pisa et al, 2012** | 1 (1M) | KD type, start, duration, disease course, survival, safety outcomes | Genetics | Effective on epilepsy, increased cholesterol and triglycerides required dietary adjustment |  |  |  |  |  |  |
| **Cederbaum et al, 1976** | 1 (1M) | KD type, survival | Genetics, KD start or duration, treatment outcomes | Ineffective on clinical state, lactate and pyruvate levels. Notice: Two sisters died at 3.5y and 6y respectively, did not receive KD |  |  |  |  |  |  |
| **McWilliam et al, 2010** | 2 (2F) | Genetics, disease course, treatment outcomes | KD type, start or duration, no treatment outcomes | Effective |  |  |  |  |  |  |
| **Group C** | | | | | | | | | | |
| **Publication** | **No of patients (M;F)** | **Available data** | **Missing data** | **KD effective/ineffective** |  |  |  |  |  |  |
| **DeBrosse et al, 2012** | 35 (unknown) | Type of KD | KD start or duration, for the treated pts: lack of genetics, survival and treatment outcomes | Ineffective/No correlation with survival or neurodevelopmental outcomes |  |  |  |  |  |  |
| **Barnerias et al, 2009** | 5 (unknown) | Type of KD | KD start or duration, genetics for the treated pts, treatment outcomes | Effective for epilepsy and paroxysmal events |  |  |  |  |  |  |
| **Kang et al, 2007** | 3 (1M;2F) | Disease course | KD type, start or duration, genetics for the treated pts, treatment outcomes | Ineffective |  |  |  |  |  |  |
| **Patel et al, 2012** | 19 (unknown) | Type of KD | KD start or duration, for the treated pts: lack of genetics, survival and treatment outcomes | Unknown |  |  |  |  |  |  |
| KD: Ketogenic Diet; PDC: Pyruvate Dehydrogenase Complex; M: male; F: female; MDK: Modified Ketogenic Diet; m: months; y: years; pts: patients | | | | | | | | | | |
