# Supplementary figures and images for: Ketogenic diet in pyruvate dehydrogenase complex deficiency: short- and long-term outcomes
Source: J Inherit Metab Dis. 2017 Jan 18;40(2):237–45. doi: 10.1007/s10545-016-0011-5 (PMC5306430; doi:10.1007/s10545-016-0011-5)

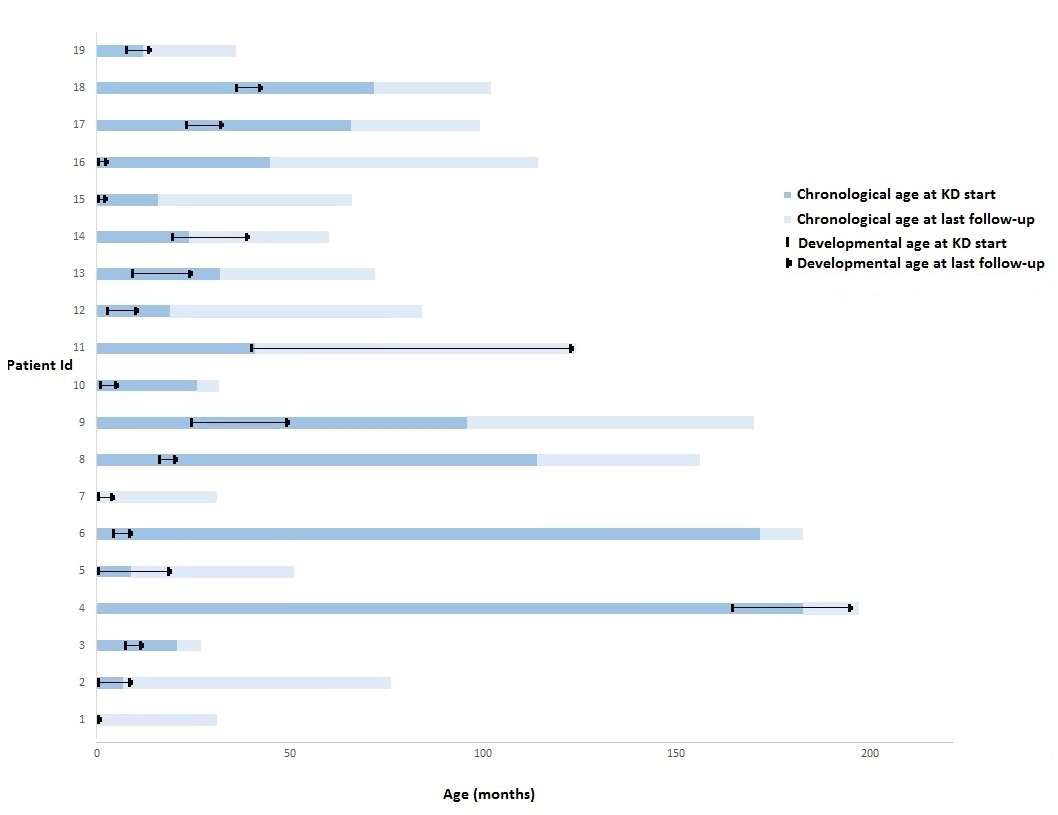

Supplement: Supplementary file 3 — Fine-motor development at baseline and during treatment (n = 19). Patient 17: At last follow-up, the patient showed improvement in executive, social, and behavioral functioning but no change in language skills. Overall neurocognitive development from baseline to last follow-up was minimally improved. Patient 18 was tested with Wechsler Scales for Children (WPPSI-III) at baseline, 6 months, and 1.5 years after diet initiation. Full-scale and verbal intelligent quotient (IQC), respectively, were as follows: 66, 66, 60 (full-scale IQ) and 78, 78, 83 (verbal IQ), with improvement in language ability, processing speed, and attention over time. Overall neurocognitive development from baseline to last follow-up was minimally improved. Patient 19: At last follow-up, the patient had developed slightly in language and social skills, with the latter corresponding to a developmental age of 19–20 months old. Overall neurocognitive development from baseline to last follow-up was minimally worse (JPG 74 kb) [file 10545_2016_11_Fig4_ESM.jpg]

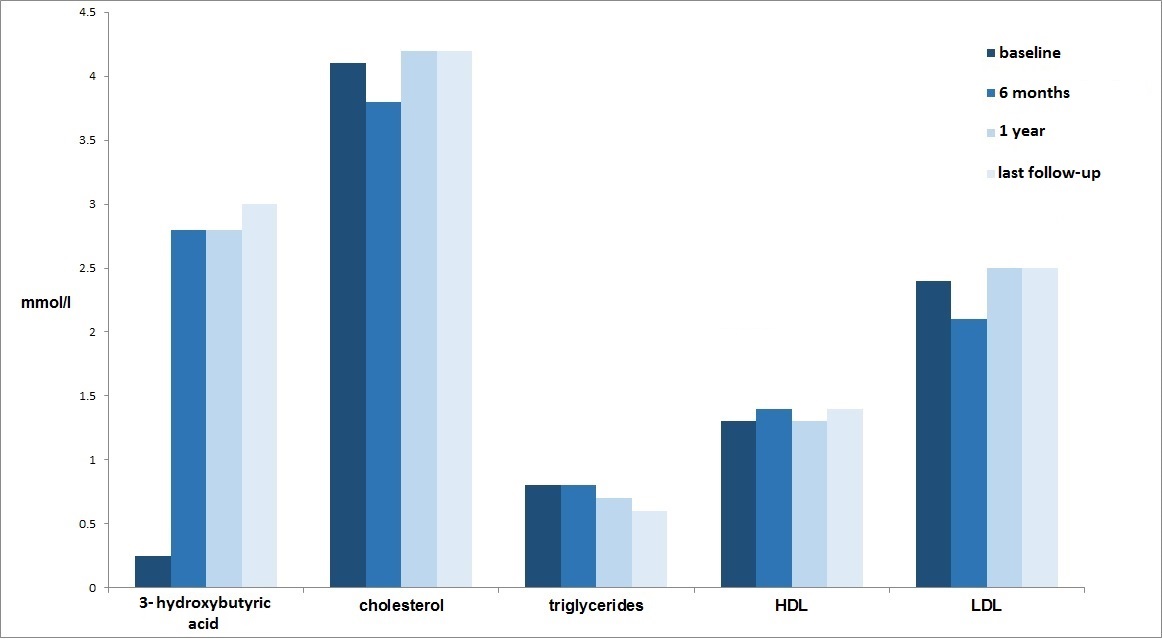

Supplement: Supplementary file 4 — Mean values of 3-hydroxybutyric acid, cholesterol, triglycerides, high-density lipoprotein (HDL), and low-density lipoprotein (LDL) in plasma at the following time points: before diet initiation (baseline), at 6 months, at 1 year, and at last follow-up (n = 19) (JPG 63 kb) [file 10545_2016_11_Fig5_ESM.jpg]
